# Multi-phase attention network for face super-resolution

**DOI:** 10.1371/journal.pone.0280986

**Published:** 2023-02-24

**Authors:** Tao Hu, Yunzhi Chen

**Affiliations:** Hangzhou Vocational and Technical College, Hangzhou, China; Taipei Medical University, TAIWAN

## Abstract

Previous general super-resolution methods do not perform well in restoring the details structure information of face images. Prior and attribute-based face super-resolution methods have improved performance with extra trained results. However, they need an additional network and extra training data are challenging to obtain. To address these issues, we propose a Multi-phase Attention Network (MPAN). Specifically, our proposed MPAN builds on integrated residual attention groups (IRAG) and a concatenated attention module (CAM). The IRAG consists of residual channel attention blocks (RCAB) and an integrated attention module (IAM). Meanwhile, we use IRAG to bootstrap the face structures. We utilize the CAM to concentrate on informative layers, hence improving the network’s ability to reconstruct facial texture features. We use the IAM to focus on important positions and channels, which makes the network more effective at restoring key face structures like eyes and mouths. The above two attention modules form the multi-phase attention mechanism. Extensive experiments show that our MPAN has a significant competitive advantage over other state-of-the-art networks on various scale factors using various metrics, including PSNR and SSIM. Overall, our proposed Multi-phase Attention mechanism significantly improves the network for recovering face HR images without using additional information.

## Introduction

Face super-resolution, a crucial component of the image super-resolution method, is the procedure that recovers the high-resolution face image from the input low-resolution face image. Since face super-resolution is a vital image restoration task and is broadly used in many situations, such as faces in surveillance videos and identity recognition, increasing attention and research focus on face super-resolution.

Recently, general super-resolution methods, including basic CNN-based methods, GAN-based methods and so on, have progressed rapidly. For example, Li Z et al. proposed a feedback method SRFBN [1], which utilizes the recurrent neural network (RNN) to form the feedback mechanism in recovering the HR face images. Zhang M et al. proposed a pixel-wised GAN named SPGAN [2], which uses a discriminative matrix and a supervised pixel-wise adversarial loss to restore realistic face images. However, these general super-resolution methods have difficulty in recovering key face structures such as eyes and mouths. These structures only make up a small part of the face, but they require more attention for the network to recover. And they are often more challenging to recover than other parts because they contain large pixel changes.

On the other hand, previous face super-resolution research mainly focuses on using additional information such as the prior and attribute information [3, 4] to reconstruct the high-resolution image. For example, Ma C et al. proposed the recursive cooperation method DIC [3], which utilizes the prior knowledge of landmark estimation to recover the face image, and uses a new attentive fusion module to improve the effect of landmark maps. Yu X et al. proposes an attribute-embedded method EFSRSA [4], which incorporates the facial attribute vectors into the autoencoder and utilizes the deconvolutional layers to upsample the feature maps. However, two main drawbacks exist: (1) More effort and computing resources are needed to obtain the additional information; (2) Effective additional information is challenging to get from the low-resolution image.

Moreover, if the face image is separated into many small parts and each part is regarded as an individual sample, we need to balance the interrelationship between parts that include key face structures and other parts which do not include key face structures, and retain the informative features. We also need to extract features from layers of different depths to recover face texture details. This suggests we need to propose a new network to solve these problems mentioned above.

Therefore, this paper introduces a Multi-phase Attention Network (MPAN), which constructs by stacking integrated residual attention groups (IRAG) for face super-resolution. The IRAG is composed of residual channel attention blocks (RCAB) [5] and an Integrated Attention Module (IAM). Each IRAG’s IAM comprises a channel attention module and a spatial attention module. The channel attention module allocates different channel weights to extract critical information. The spatial attention module assigns a distinct weight to each position of the feature map. The IAM makes the network place a greater emphasis on important components such as the eyes and mouths. Our MPAN also applies the concatenated attention module (CAM) to weight feature layers of different depths. The concatenated attention module makes the network focus more on the informative layer, not the deeper layer of the network, leading the network more efficient in recovering face textures details. For this paper, the main contributions are as follows:

1. We propose a multi-phase attention network (MPAN) without relying on prior or attribute information. And our MPAN has an advantage over other face super-resolution methods in recovering face HR images. Also, it achieves better performance than other networks on various scale factors with metrics including PSNR and SSIM [6, 7].
2. We propose a novel IRAG structure, which is the basic block of the network to construct the deep network. And the IRAG structure bootstraps the face structures, for example, face outline.
3. By stacking IRAG in the MPAN, the IAM in different IRAG makes the network focus more on key face structures such as eyes and mouths.
4. The proposed CAM makes the network more efficient in recovering face texture details and greatly improves the representation of our MPAN.
5. The proposed IAM and CAM form the multi-phase attention to reallocate features among channels, positions, and layers. Extensive experiments show the great advantage of our MPAN.

## Related work

### Face super-resolution methods

There are five categories of face super-resolution methods [8]. General face super-resolution methods concentrate on effective networks with various advanced structures such as residual block [9–11] and attention mechanisms [12] to enhance the effectiveness. General face super-resolution methods can be further subdivided into four distinct groups: basic CNN-based methods, GAN-based methods, reinforcement learning-based methods, and ensemble learning-based methods. Prior-based face super-resolution methods first extract the prior facial information such as facial heatmaps [13], facial landmarks [14], and facial parsing maps. Then utilize them to reconstruct a clearer facial structure. Identity-preserving face super-resolution methods take full advantage of the identity information of face images to maintain identity consistency. Attribute-constrained face super-resolution fully exploits the facial semantic knowledge, for example, the description by the witness. Reference face super-resolution methods utilize several high-resolution face images of the low-resolution face image. These high-resolution images may be used to restore facial photographs by providing identification information. But sometimes, it is tough to find the available reference image.

In recent decades, various face super-resolution methods have been presented. The pioneer CNN-based method for super-resolution was EDSR [15] which can also be applied in the face super-resolution. EDSR removes an unnecessary batch normalization module from the residual blocks to improve performance. Based on the EDSR, Haris M et al. first proposed the DBPN [16] which uses the deep back-projection to enhance the interdependence between low and high-layer image features. Zhang Y et al. first introduced the RDN [17] which uses dense connections to learn more effective features from the previous parts. The previously mentioned three methods mainly focus on residual blocks and skip connections. Attention mechanisms are also applied in face super-resolution. Chen Y et al. proposed a face image super-resolution method [18], which applies the channel attention mechanism on feature maps. This method extracts features from LR images, reallocates channel features, and recovers HR images at various scales. The latest proposed methods restore the high SR from the low SR image base on extra facial information such as facial landmarks, facial heatmap, and facial parsing maps. Chen C et al. proposed a progressive semantic-aware style transformation method PSFR-GAN [19], which takes advantage of the parsing maps and pixel space features from LR face images. This method also has a semantic aware loss function that computes the semantic region loss to recover the face key structures better. However, the mentioned methods either lead to the loss of key face structure details in intermediate feature layers [20] because of the very deep depth or generate some artifacts or unreal details. We propose a multi-phase attention network that contains the concatenated attention module and the integrated attention module to reallocate the features across layers, channels, and positions.

### Attention network

Various networks that use attention mechanisms are introduced to solve the vision tasks. The key point of the attention mechanism is to utilize the attention map to reweight features in the network [21]. Hu J et al. first proposed a squeeze and excitation network (SENet) [22], which introduced attention mechanisms and modularity first compared to other networks. Wang Q et al. proposed a Efficient Channel Attention for Deep Convolutional Neural Networks (ECA-Net) [23], which uses the convolution operation to generate the channel attention map. Woo S et al. proposed a convolutional block attention module (CBAM) [24], which uses spatial attention and channel attention in sequence. Park J et al. proposed a Bottleneck Attention Module (BAM) [25], which consists of spatial attention and channel attention and places them in two separate paths. Mugeet A et al. proposed a Multi-Attentive Feature Fusion Super-Resolution Network (MAFFSRN) [26], which constructs by stacking muti-attention blocks. The multi-attention block integrates and enhances channel and spatial attention. Zhang Y et al. proposed the residual channel attention network (RCAN) [5], which applies not only residual structure with long skip connections but also channel attention to allocate weight to different channels. Dai T et al. proposed a second-order attention network (SAN) [27], which combines long-distance interdependences with the entire network structure information and obtains remarkable performance. Wang C et al. proposed a two-step face super-resolution network (FishSRNet) [28], which utilizes face prior knowledge. And the FishSRNet uses multi-scale channel mechanisms and spatial attention mechanisms. Zhao H et al. proposed a lightweight and effective network (PAN) [29], which is composed of a novel pixel attention mechanism. Pixel attention works the same way as channel attention and spatial attention. But it applies the 3D attention maps and has fewer parameters. However, these previously mentioned algorithms only focus on the channel and spatial interdependences and neglect the correlations between layers of different depths. As a result, we propose the multi-phase attention network to fully utilize the feature interrelations between hierarchical layers.

## MPAN for face super-resolution

### Ethics statement

**Fig 1:**
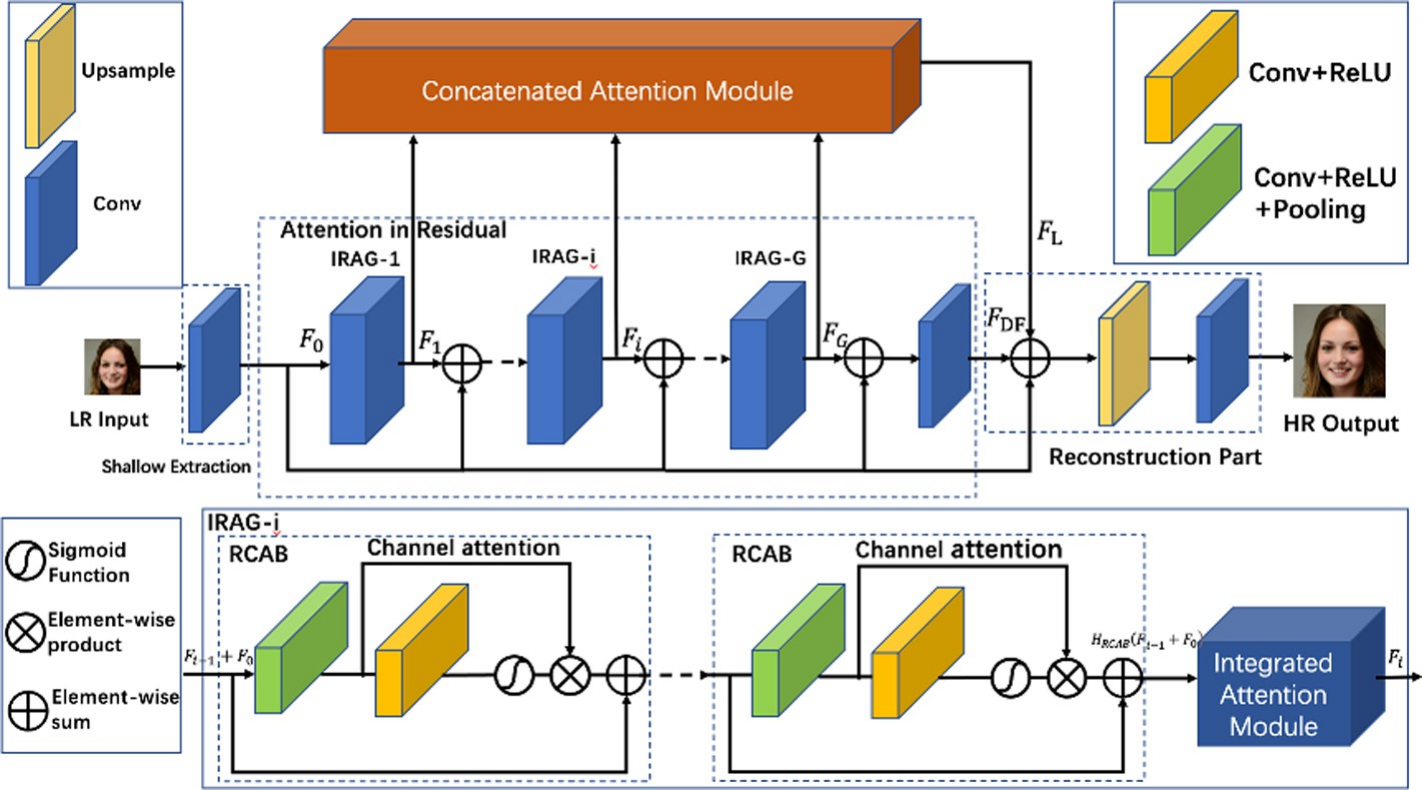
The network architecture of our MPAN.

The individuals pictured in Figs 1, 5–9 have provided written informed consent (as outlined in PLOS consent form) to publish their image alongside the manuscript.

### Network architecture

As illustrated in Fig 1, our proposed MPAN is primarily composed of four major components: shallow feature extraction, attention in residual (AIR), concatenated attention module (CAM) and the final reconstruction part.

#### Shallow feature extraction

We refer to *I*_*LR*_ and *I*_*SR*_ as the input and output of MPAN. As investigated in [15, 30], we extract the shallow feature *F*_0_ from the LR input using a convolutional layer:

$$F_{0}=H_{SE}(I_{LR})$$ (1)

Where the *H*_*SE*_ represents the convolution operation. Then the *F*_0_ is utilized as the input of the backbone attention in residual (AIR).

#### Attention in residual (AIR)

The details of our AIR structure are shown in Fig 1. The AIR structure consists of *G* integrated residual attention groups (IRAG) and *G* skip connections (SC). Every IRAG further consists of *B* residual channel attention blocks (RCAB) [5] and an Integrated Attention Module (IAM). Simply stacking many IRAGs may lead to bad performance. So the skip connections (SC) are used to stabilize the training of the deep network [31]. The function of the first IRAG in the AIR structure can be represented as:

$$F_{1}=H_{1}(F_{0})$$ (2)

And the other IRAG in the AIR structure can be represented as:

$$F_{i}=H_{i}(F_{i-1}+F_{0}),\;i=2,3,\;\ldots ,G,$$ (3)

*F*_*i*_ denotes the *i*-th IRAG’s output. *G* is the number of IRAG. And *H*_*i*_ is the function of the *i*-th IRAG. So the output of AIR structure is formulated as:

$$F_{DF}=W_{CF}(F_{G}+F_{0})$$ (4)

*F*_*G*_ is the output of the last IRAG. And *F*_*DF*_ is the output of the AIR structure. The *W*_*CF*_ is the weight assigned to the Conv layer at the AIR’s tail.

#### Concatenated attention module (CAM)

Previous AIR structure extracts the hierarchical features *F*_*i*_. To model the feature correlations between layers, we further propose a concatenated attention module (See Section3.2 in detail) that weights the different layers of depths.

The proposed concatenated attention fully utilizes features from all previous layers and is formulated as:

$$F_{L}=H_{CA}(F_{1},F_{2},\ldots ,F_{i},\ldots ,F_{G}),\;i=1,2,\ldots ,G,$$ (5)

where *H*_*CA*_ denotes the CAM. The CAM scales the fused intermediate features *F*_*i*_ which are generated by output features of IRAG. Consequently, high-contribution feature layers in CAM are enhanced, while redundant feature layers are suppressed.

#### Image reconstruction

After processing features in the previous structure, we perform an element-wise summation to combine output features. To convert the scale sampling, we next apply the sub-pixel convolution, which acts as the upsampling module. The sub-pixel convolution utilizes an array of upscaling filters to convert LR feature maps to HR output, reducing the upscale operation’s computational complexity. Then the upscaled features are then performed a convolution operation. The whole process can be represented as:

$$I_{SR}=H_{REC}(F_{0}+F_{L}+F_{DF})$$ (6)

Where the *H*_*REC*_ represents the reconstruction operation which includes the sub-pixel convolution and the convolution operation. The inputs of the *H*_*REC*_ are *F*_0_ + *F*_*L*_ + *F*_*DF*_. The *I*_*SR*_ is the operated SR output.

### Concatenated attention module

Although dense skip connections are used to learn more effective features from shallow layers and stabilize the training of the deep network [10], the interdependences between layers are not fully utilized. Thus, we propose a novel CAM that learns the interrelationship of layer features of various depths to enhance the performance of network representation. Specifically, the CAM forms feature maps of feature groups into two vector matrices and constructs interdependencies between different feature layers. The CAM allocates distinctive attention weight to the layer features, improving the feature representation capability.

**Fig 2:**
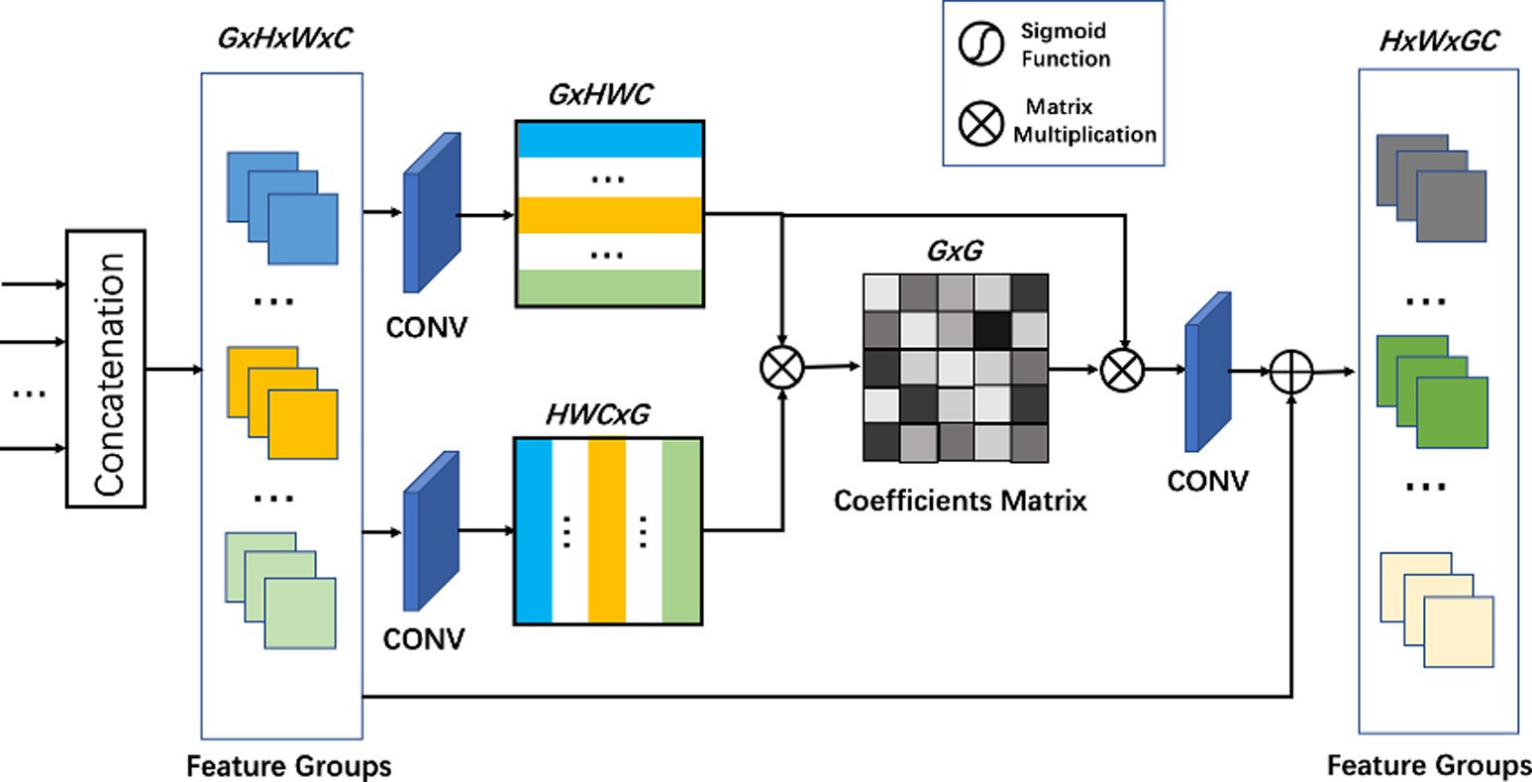
The structure of the proposed concatenated attention module.

Fig 2 depicts the structure of the CAM. The input consists of discrete feature groups taken from *G* integrated residual attention groups(IRAG). Then we do the concatenation operation. The dimension of the concatenation output is *G* × *H* × *W* × *C*. Then, we restructure the feature groups into two 2D matrices using convolution. One matrix’s dimensions is *G* × *HWC*. The other is *HWC* × *G*. Also, we do the matrix multiplication to the previous two matrices to calculate the interdependencies of different feature layers

$$w_{ij}=\varphi \left(\theta {\left(FG\right)}_{i}\cdot {\left(\theta (FG)\right)}_{j}^{T}\right),\;i,j=1,2,\ldots ,G,$$ (7)

Where *φ*(∙) and *θ*(∙) represent the softmax and reshape operation. *i* denotes the *i*-th row of reshaped feature groups matrix. *j* denotes the *j*-th column of reshaped transpose feature groups matrix. *w*_*ij*_ denotes the corresponding coefficients between *i*-th and *j*-th extracted feature layers. Next, we multiply the calculated coefficient matrix with the first reshaped feature groups matrix using matrix multiplication:

$$F_{ij}=\sum_{k=1}^{N}w_{ik}FG_{kj},\;i,j=1,2,\ldots ,G,$$ (8)

Where *FG*_*kj*_ denotes the *k*-th row *j*-th column element of the feature groups matrix. *F*_*ij*_ denotes the *i*-th row *j*-th column element of output of the matrix multiplication. At last, we reshape the output of the matrix multiplication and add the input feature groups. Overall, the proposed CAM structure makes the network concentrate on more informative and intermediate layer features.

### Integrated attention module

The existing channel attention networks [22, 23] construct a weight correlation matrix and allocate different weights to channels, with little consideration of the spatial features. On the other hand, spatial attention networks [24, 25] neglect the distinctive weights of channels. As a result, we propose an integrated attention module (IAM) that fuses the channel and spatial attention modules to increase accuracy.

Every IRAG consists of *B* residual channel attention blocks (RCAB) [5] and an Integrated Attention Module (IAM). The input of the first IRAG is *F*_0_. And the output features of RCABs in the first IRAG are *H*_*RCAB*_(*F*_0_). The output of the first IRAG is *F*_1_. Here we mainly focus on the condition *i* ≥ 2. So the input of the *i*-th IRAG is *F*_*i*-1_ + *F*_0_. And the output features of RCABs in the *i*-th IRAG are *H*_*RCAB*_(*F*_*i*-1_ + *F*_0_). *H*_*RCAB*_ represents the function of *B* residual channel attention blocks (RCAB). We perform the IAM for the output features of the RCAB, as shown in Fig 1.

**Fig 3:**
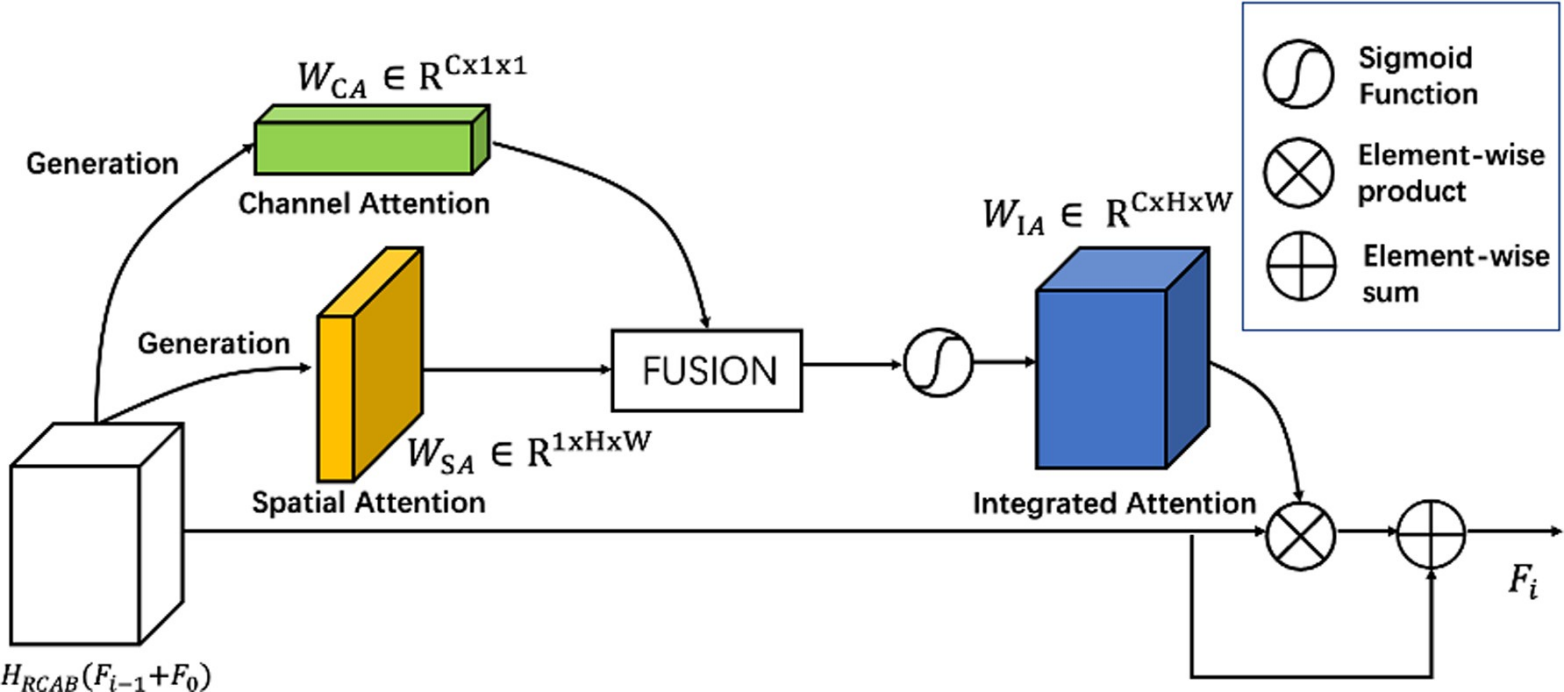
The structure of the proposed integrated attention module.

The structure of our proposed IAM is shown in Fig 3. We perform the channel attention operation to obtain the channel attention weight *W*_*CA*_. The dimension of *W*_*CA*_ is *C* × 1 × 1. This process contains two fully connected layers and a batch normalization operation which can be represented as:

$$W_{CA}=BN(FL(FL(H_{RCAB}(F_{i-1}+F_{0}))))$$ (9)

Where the *FL* represents the fully connected layer. And the *BN* represents the batch normalization operation. We also perform the spatial attention operation to generate the spatial attention weight *W*_*SA*_. The dimension of *W*_*SA*_ is 1 × *H* × *W*. This process contains two convolutions and two dilated convolutions which can be represented as:

$$W_{SA}=W_{conv}(W_{dconv}(W_{dconv}(W_{conv}(H_{RCAB}(F_{i-1}+F_{0})))))$$ (10)

We replicate the number of the channel attention weight *W*_*CA*_ to *HW*. And rearrange these channel attention weights to get a new channel attention weight whose dimension is *C* × *H* × *W*. At the same time, we replicate the number of the spatial attention weight *W*_*SA*_ to *C*. And stack these spatial attention weights to get a new spatial attention weight whose dimension is *C* × *H* × *W*. Then we do the element-wise summation to fuse [32] two new attention weights and get the *W*_*IA*_. Moreover, we do element-wise multiplication with the feature maps *H*_*RCAB*_(*F*_*i*-1_ + *F*_0_) and the correlation weights matrix *W*_*IA*_. At last, we add the feature map *H*_*RCAB*_(*F*_*i*-1_ + *F*_0_) with the output of the element-wise multiplication to obtain the weighted features:

$$F_{i}=H_{RCAB}(F_{i-1}+F_{0})⊙W_{IA}+H_{RCAB}(F_{i-1}+F_{0}),$$ (11)

Where ⨀ denotes the element-wise multiplication. Thus, *F*_*i*_ is the weighted operated output performed by the integrated attention module. Unlike the traditional channel attention and spatial attention module, our IAM selectively learns the inter-channel and in-channel features by constructing a channel and spatial adjusted independent weights intercorrelation.

### The proposed algorithm of MPAN

**Fig 4:**
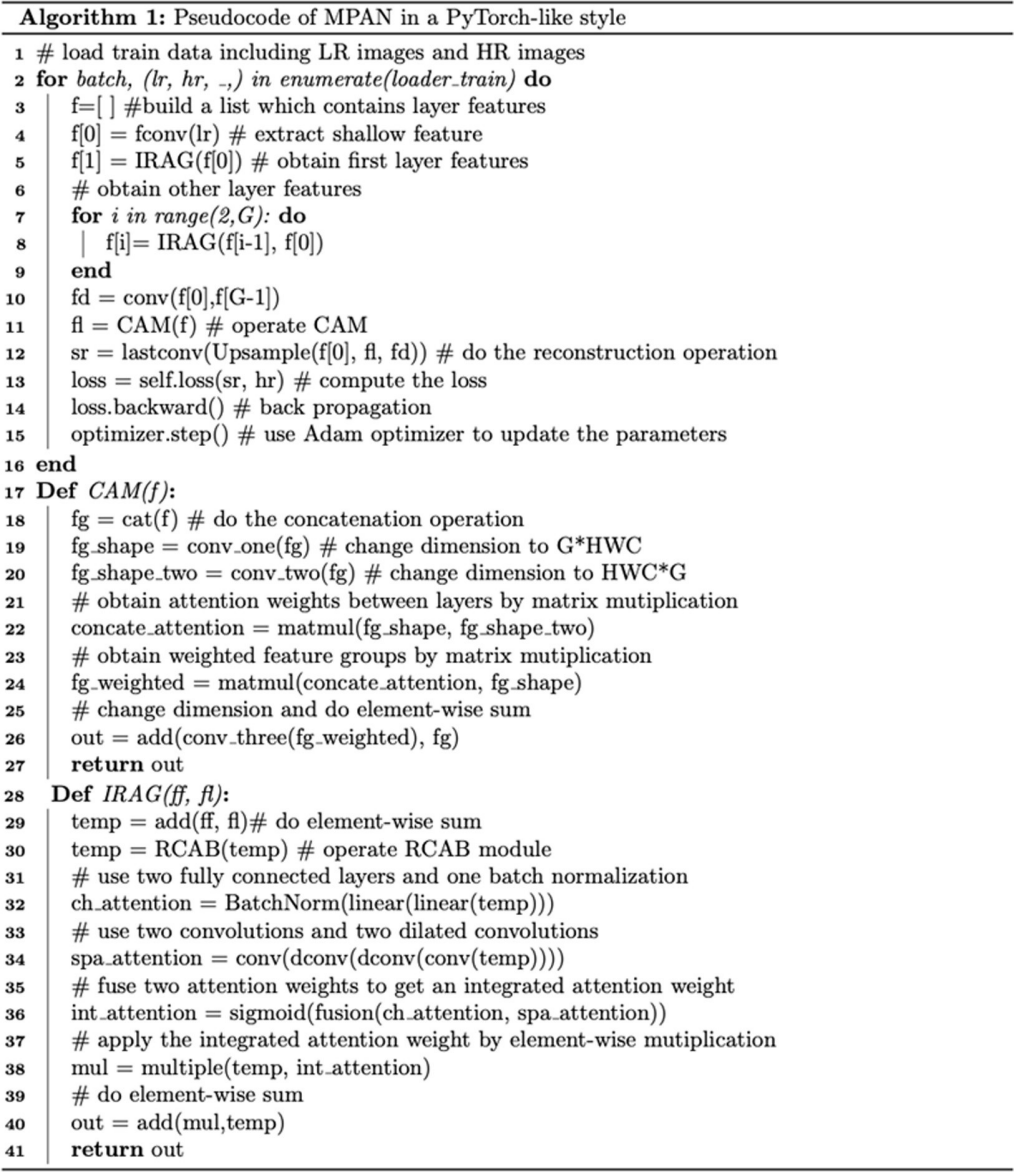
Pseudocode of MPAN in a PyTorch-like style.

The Fig 4 provides the pseudocode of MPAN in a PyTorch-like style. This algorithm includes three parts: the main part, the CAM function and the IRAG function. The main part provides the code for one batch data training including forward propagation, loss computing [33] and back propagation. The CAM function provides the code for concatenation attention construction and application. The IRAG function provides the code for RCAB modules and integrated attention construction and application.

## Experiments

This section begins by analyzing the contributions of the two proposed attention modules. Then we compare our MPAN algorithm to the most advanced algorithms using our test dataset. Results on more images are shown in the additional part.

### Settings

#### Datasets

The FFHQ [34] is a high-quality human faces dataset. It is composed of 7000 high-quality face images that were downloaded in 1024x1024 resolution from the internet and used for various human face tasks such as human face detection and super-resolution. We create our dataset by ourselves. As the training set, choose 22,000 photos of individuals of varying ages and sexes from the FFHQ dataset. Additionally, choose 2000 images for the testing set. The face images in the training set and testing set are entirely distinct [35]. We resize the images to 128x128 with bicubic interpolation operation [36] as the ground-truth HR images. We transform the restored RGB result into YCbCr space. The final results are assessed on metrics such as PSNR and SSIM [37] on the luminance Y channel [38].

#### Implementation details

We use PyTorch [39] platform to implement the multi-phase attention network. In our experiment, the patch size is set as 96 x 96. Our network is trained with ADAM [40] optimizer. The batch size [41] is 16. The initial learning rate [42] is set as 10^−4^ and the learning rate decay factor is 0.5 after each of 2 * 10^5^ iterations. We use data augmentation [43] which randomly rotates the training images by 90°, 180°, 270° and horizontal flipping to avoid overfitting. We apply attention in residual (AIR) as the main part of our network. The number of integrated residual attention group (IRAG) in AIR is *G* = 10. And there are *B* = 20 residual channel attention blocks (RCAB) in each IRAG. The number of epochs is set as 200. Every result requires two days of training on an Nvidia GTX 3080Ti GPU.

### Ablation study regarding the proposed CAM and IAM

The proposed CAM and IAM generate weight correlations matrix on different layers, channels, and spatial. But we need to know the real effects of our two modules. We conduct experiments without using CAM and IAM on our face dataset with a scale factor of 4x and make comparisons.

**Fig 5:**
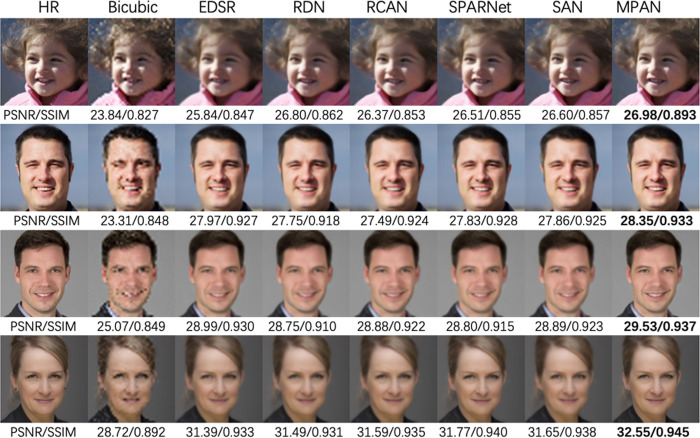
Visual comparison for X3 SR on our face dataset. The best results are bold. Our network achieves superior performance and recovers more face details than previous networks.

Table 1 presents the quantitative results. The model with CAM achieves 0.06 dB greater PSNR performance than the baseline model without the CAM and IAM modules, whereas the model with IAM simulates 0.16 dB. The model using both CAM and IAM modules performs best on PSNR on scale factor X4. Fig 5 represents the vital effectiveness of face structures and details using both CAM and IAM modules.

**Table 1:** Ablation study about the proposed CAM and IAM.

	baseline	with CAM	with IAM	CAM And IAM
**PSNR/SSIM**	24.373/0.8381	24.433/0.8382	24.533/0.8383	24.573/0.8386

### Ablation study regarding the number of integrated residual attention group

In this paper, the integrated residual attention group (IRAG), the main part of the network, is composed of residual channel attention blocks (RCAB) and the IAM. The IAM integrates channel attention and spatial attention to promote the network representation ability. We study the effect of IRAG numbers in MPAN. Specifically, we set the IRAG number equal to one, three, five, and ten. And the PSNR and SSIM results on scale factors X4 are shown in Table 2. The evaluation is apparently improved with the increase of IRAG numbers. This ablation study demonstrates the significant impact of IRAG modules. So we choose option ten IRAG which obtains the best scores as part of our network.

**Table 2:** Ablation study about IRAG numbers.

	MPAN(1 IRAG)	MPAN(3 IRAG)	MPAN(5 IRAG)	MPAN(10 IRAG)
**PSNR/SSIM**	24.430/0.8375	24.461/0.8380	24.543/0.8383	24.573/0.8386

### Ablation study regarding the number of residual channel attention block

We conduct an ablation research to determine the optimal number of residual channel attention blocks (RCAB) to feed to the proposed integrated residual attention group (IRAG) module. Specifically, we apply five, ten, fifteen and twenty RCAB in each IRAG module and evaluate our network on the testing dataset. As indicated in Table 3. we compare our four types of models based on the X4 scale factor. Clearly, when the number of RCAB increases, the PSNR and SSIM values on test data increase as well. This ablation study illustrates the effectiveness of RCAB. So option twenty RCAB, which has the best performance, is our choice for the network.

**Table 3:** Ablation study about RCAB numbers.

	MPAN(5 RCAB)	MPAN(10 RCAB)	MPAN (15 RCAB)	MPAN (20 RCAB)
**PSNR/SSIM**	24.453/0.8377	24.473/0.8380	24.554/0.8384	24.573/0.8386

### Comparisons with state-of-the-art methods

We compare our proposed network with state-of-the-art methods, including SR methods like EDSR [15], RDN [17], RCAN [5], SPARNet [44], and SAN [27]. We utilize the open-source code of the models above and train them using the same dataset.

#### Overall results

**Fig 6:**
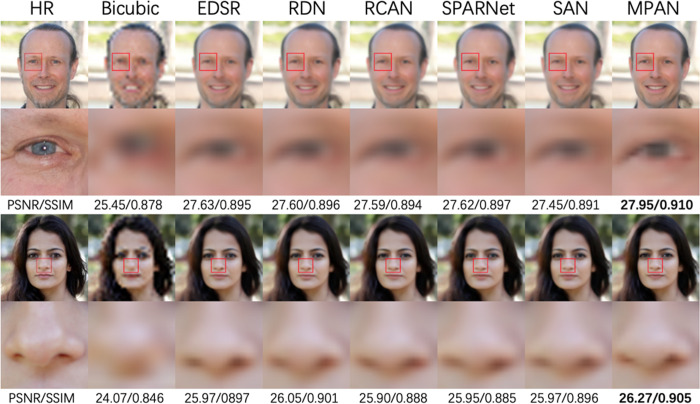
Visual comparison for 4X SR on our face dataset. The best results are bold.

Quantitative comparisons of X2, X3, and X4 on our face image dataset are shown in Table 4. In terms of PSNR and SSIM scores, our MPAN performs better than other state-of-the-art approaches. With two proposed attention modules, our MPAN recovers face details structure better. It is shown in Fig 6 that most other state-of-art methods are unable to recover the nose and eyes accurately and suffer from blurring details. But MPAN obtains shaper results similar to the ground truth HR images.

**Table 4:** Quantitative results with 128x128 output and scale factor of X2, X3 and X4. The top and second-place results are emphasized in bold and underlined, respectively.

Methods		DBPN	EDSR	RDN	RCAN	SPARNet	SAN	MPAN
**PSNR**	**X2**	28.529	28.800	28.796	28.784	28.805	28.831	**28.977**
**SSIM**		0.9265	0.9296	0.9294	0.9292	0.9298	0.9301	**0.9318**
**PSNR**	**X3**	24.802	25.427	25.174	25.125	25.301	25.314	**25.553**
**SSIM**		0.8509	0.8643	0.8592	0.8590	0.8601	0.8618	**0.8672**
**PSNR**	**X4**	24.109	24.549	24.482	24.512	24.522	24.411	**24.573**
**SSIM**		0.8258	0.8380	0.8371	0.8372	0.8375	0.8329	**0.8386**

#### Detailed comparisons

**Fig 7:**
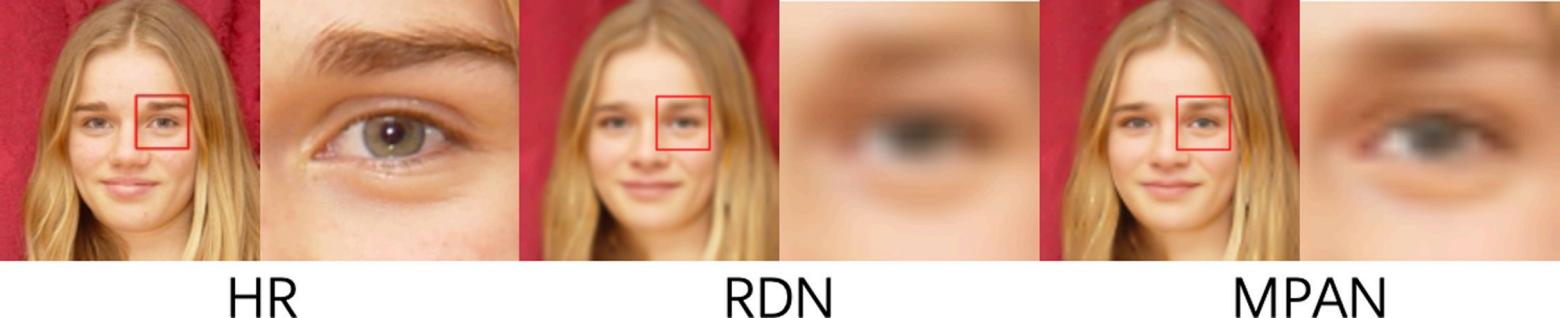
Comparative results of MPAN and RDN for 4X SR.
- *Compared with SPARNet*. The SPARNet is composed of stacking Face Attention Units(FAUs) which consist of the attention branch and the feature branch. The attention branch utilizes the spatial attention mechanism to focus more on feature-rich face regions. However, our MPAN integrates the channel and spatial attention mechanism to form the integrated attention mechanism, making the network pay more attention to the key face structures. Also, our MPAN rescales the features of layers to form concatenate attention mechanism, allowing the network to reconstruct facial texture information. So our MPAN performs better than SPARNet shown in Table 4 and Fig 8.

- *Attention mechanisms*. RCAN, SPARNet, SAN, and MPAN are the methods being compared that involve attention mechanisms. RCAN is designed and widely used for various SR tasks. It is comprised of a channel attention mechanism that adaptively rescales channel-wise features by assigning distinct channel weights. Channel attention has been proven to be effective for conventional SR tasks, but spatial attention is substantially more advantageous when addressing low-resolution face super-resolution issues. This is why the other three methods, which contain spatial attention mechanisms, perform better than RCAN shown in Table 4.
- *Compared with RDN*. The RDN makes full use of hierarchical features from layers of different depths by using the residual dense block which includes dense connections from lower to higher layers. The RDN does not use any attention mechanism. In contrast, our MPAN uses not only the layer, channel, and spatial attention mechanisms but also skip connections. Our MPAN outperforms RDN on evaluation metrics and recovering key face structures shown in Table 4 and Fig 7, demonstrating the attention mechanism’s superiority.

**Fig 8:**
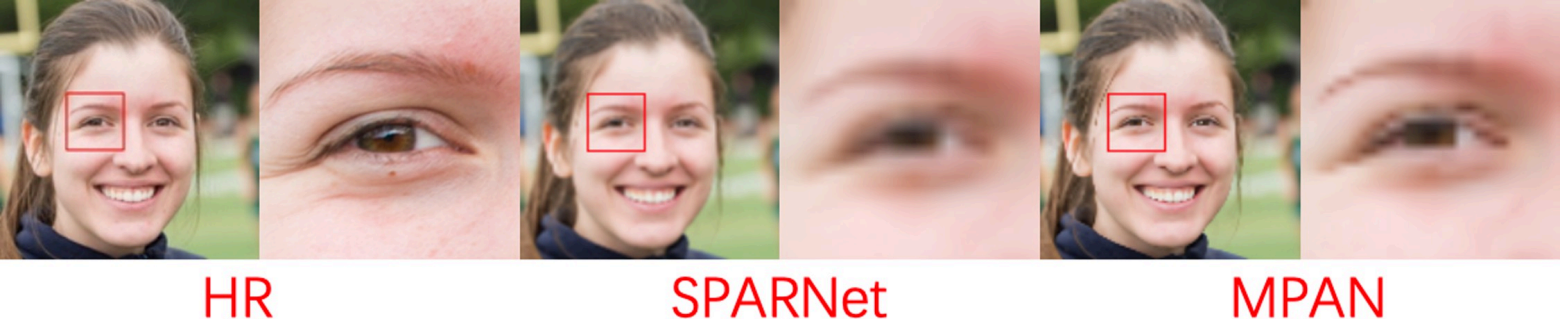
Comparative results of MPAN and SPARNet for 2X SR.
- *Compared with SAN*. The SAN uses both the channel and spatial attention mechanism. It places the spatial attention module at the front and end of the network and places the channel attention module in every part of the network backbone. Our MPAN also uses layer, channel, and spatial attention mechanisms. But the specific implementation and usage are different. Our MPAN places the channel and spatial attention mechanism in each part of the network. And use the layer attention mechanism to allocate different weights to layers, which is beneficial to restoring texture details. Thus, our MPAN outperforms SAN on all upscale factors clearly shown in Table 4. Our MPAN can recover more face details than SAN shown in Fig 9.

**Fig 9:**
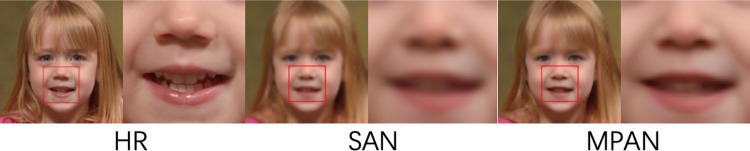
Comparative results of MPAN and SAN for 4X SR.
- *Compared with different upscale factors*. Our models are trained using X2, X3, and X4 upscale factors. The X2 model was trained from scratch, while the X3 and X4 model were initialized with the pre-trained X2 model. We compare the SR results of a different upscale factor in Table 4. MPAN achieves the most significant outcomes for all upscale factors, particularly the X2 upscale factor. Among all upscale factors, all the models obtain the best results for the X2 upscale factor and the worst ones for the X4 upscale. So the models can reconstruct face images better with enough face features.

## Conclusions

In this paper, we build the Multi-phase Attention Network (MPAN) for face super-resolution, which rescales the features among different layers, channels, and positions. Specifically, The integrated residual attention groups (IRAG) build the basic block of the MPAN. The concatenated attention module (CAM) reallocates dependencies among layers of different depths. The integrated attention module (IAM) incorporates features of channels and positions. The above two attention modules form the multi-phase attention to further enhance the performance of recovering face HR images. The extensive experiment demonstrates that our MPAN performs better than other state-of-the-art methods in terms of PSNR and SSIM, making the restored SR face images more realistic to the real HR face images.
